# Effect of UVA1 on hypertrophic scarring in the rabbit ear model

**DOI:** 10.1042/BSR20190007

**Published:** 2020-01-21

**Authors:** Tong Zhang, Zhiming Shen, Jie Zheng, Rui Jiang

**Affiliations:** 1 Department of Dermatology, Minhang Hospital, Fudan University, Shanghai 201109, China; 2 Department of Experimental Animal Science, Shanghai Jiao Tong University School of Medicine, Shanghai, China; 3 Department of Dermatology, Rui Jin Hospital Affiliated to Shanghai Jiao Tong University School of Medicine, Shanghai, China; 4 Department of Orthopaedics, Gongli Hospital of Shanghai Pudong New Area, Shanghai 200135, China

**Keywords:** a-smooth muscle actin, Hypertrophic scar, Matrix metalloproteinase-1, Rabbit model, Tissue growth factor-β, Ultraviolet A1

## Abstract

Hypertrophic scars (HTSs) are common and cause functional and psychological morbidity. UVA1 (340–400 nm) phototherapy has been previously shown to be effective in the treatment of localized scleroderma, systemic sclerosis, and POEMS syndrome with minimal side effects, all of which are presented as collagen fibrils hyperplasia that is common with scarring in skin histology. ***In the present study***, we aimed to investigate the impact of UVA1 on the protein expression of TGF-β signal pathway and myofibroblasts in a rabbit model of cutaneous scarring. Full-thickness skin wounds (2 cm × 5 cm in diameter) were made in New Zealand white rabbits to establish the hypertrophic scarring model. New Zealand white rabbits were divided into two treatment groups (*n*=30 wounds per group with an equal number of controls): medium-dose of UVA1 phototherapy group: 60 J/cm^2^; high-dose of UVA1 phototherapy group: 110 J/cm^2^. Left ears were used for treatment and the right ones were used for control. Treatment was administered five times weekly for 6 weeks. Treated and untreated control wounds were harvested at various time points and examined by histologic examination, immunohistochemical assessment, and ultrastructural evaluation. The results showed that UVA1 phototherapy caused a significant reduction in dermal thickness by histological features, whereas the scar index was descended significantly in both medium- and high-dose UVA1 groups compared with the control group. Examination of immunohistochemistry also revealed a marked suppression of tissue growth factor-β (TGF-β) (both medium- and high-dose), α smooth muscle actin (α-SMA) (only high-dose), and tissue inhibitor of metalloproteinase 1 (TIMP-1) (only high-dose), and apparent increase in matrix metalloproteinases (MMP-1) (both medium- and high-dose) compared with the control. The ultrastructural evaluation showed the collagen fibers’ diameter had shrunk, and that fibroblastic cytoplasm was not affluent and in a quiescent stage. These findings of the present study suggested that administration of UVA1 irradiation is effective to improve the experimental HTS model and raises a possibility of the therapeutic approach of UVA1 in the scar. Although not directly examined in the present study, MMP inhibition is hypothesized to be responsible for this effect. However, early UVA1 treatment could not prevent the formation of scar model.

## Introduction

Keloids and hypertrophic scars (HTSs) are benign hyperproliferative growths of dermal collagen. [1]. They are common problems affecting 4.5–16% of the general population and are a common subject of dermatologic consultations [2]. Keloids and HTS often cause unacceptable disfigurement to the affected individuals. In addition, they may be painful or pruritic, and restrict range of motion [3,4]. Therapeutic management of such conditions remains challenging due to their high rate of recurrence and lack of curative treatment [5]. Several treatments, such as intralesional injection of various agents (e.g. corticosteroids and/or 5-fluorouracil, interferon, bleomycin), surgical removal, occlusive dressing, cryotherapy, radiotherapy, laser therapy, and silicone gel sheeting had been used either alone or in various combinations, some with potential side effects and varying degrees of success [6,7]. Intralesional steroid injection, the long-term standard of keloid therapy, has been used most commonly. Whereas, steroids cause a lot of contraindication and adverse reactions, physical treatment has been paid close attention gradually. The introduction of UV therapy has substantially changed the therapeutic possibilities of HTSs, particularly in providing new insights into the pathogenesis of the disease.

Recently, UVA1 (340–400 nm) phototherapy has received much more attention as a possible treatment for atopic dermatitis [8], morphea [9], and scleroderma [10,11]. The advantages of UVA1 include deep penetration into the skin, which allows treatment of deeper tissues, and less DNA absorption than that which occurs with shorter, more erythemogenic UV radiation. Thus, UVA1 therapy offers a theoretical decrease in the risk of burning and cancer formation. As is known, HTS are characterized by excessive deposition of collagen in the dermis and subcutaneous tissues secondary to traumatic or surgical injuries. Although the mechanism of keloid and HTS formation have not been fully delineated, it has been postulated that keloid- and HTS-derived fibroblasts produce higher amounts of collagen per cell compared with normal fibroblasts [12]. Focal release of cytokines are supposed to play a key role in fibroblast proliferation and collagen synthesis. In particular, tissue growth factor-β (TGF-β) is supposed to play a crucial role in tissue fibrosis. Multiple action of TGF-β includes the strong induction of extracellular matrix (ECM) deposition by stimulating the production of matrix proteins, inhabiting proteases that degrade matrix, and modulating the expression of matrix receptors on the cell surface. Matrix metalloproteinase-1 (MMP-1), as well as its specific inhibitor, tissue inhibitor of metalloproteinase-1 (TIMP-1) also play significant roles in collagen degradation. Furthermore, myofibroblasts expressed by α-smooth muscle actin (α-SMA) also affect the HTS formation and evolution. Thus, suppression of the overwhelming and uncontrolled fibroblast activity in keloid and HTS may be essential in therapeutic approaches to these abnormal wound responses [13]. Given the *in vitro* work regarding the effect of UVA1 on stimulated collagenase production by fibroblasts, we decided to use UVA1 phototherapy to treat the HTSs in spite of the fact that studies on HTS treated by UVA1 do not comprise more than several cases’ reports. We studied the success of our treatment on the animal model of HTS in this challenging clinical scenario. The possibility of HTS treated by UVA1 phototherapy was an attractive option.

HTS is a fibroproliferative disorder that results in excessive deposition of collagen and other ECM molecules following damage to the deep dermis by thermal injury or other forms of trauma, and often causes severe cosmetic and functional impairment [14,15]. Although the molecular and cellular events that lead to HTS have been studied extensively, the pathogenesis of this condition is still not well understood. Patients that suffer from HTS, especially in visually sensitive areas of the body, seek to recover from this ugly appearance and associated symptoms as soon as possible with atraumatic or less invasive ways.

UVA1 (340–400 nm) has been recently recognized as an excellent treatment option for treatment of atopic dermatitis, morphea, and scleroderma. Several clinical studies have shown that UVA1 phototherapy is an effective treatment of keloids and HTSs [16,17]. Encouraged by the positive results of UVA1 irradiation on localized scleroderma, Asawanonda et al. [18], Hannuksela-Svahn et al. [19], and Polat et al. [20] successively tried to use high-dose UVA1 phototherapy on keloid scars. Asawanonda et al. [18] and Polat et al. [20] obtained satisfactory outcome, but results of Hannuksela-Svahn et al. [19] were unsatisfactory. He considered the lower single and accumulated dose as the possible reason for treatment failure.

We developed a reproducible and quantifiable animal model of hypertrophic scarring in a rabbit ear that has shown to behave similarly to human hypertrophic scarring. The purpose of the present study was to investigate the effect of UVA1 on the rabbit ear HTS model, and further, to observe whether UVA1 phototherapy could prevent scar formation.

## Materials and methods

### Rabbits

New Zealand white rabbits (female) purchased from Shanghai Laboratory Animal Research Center weighing approximately 2.5 kilogram were used. Rabbits were kept in separate, clean rooms in the Animal Facility of Shanghai Jiao Tong University School of Medicine. These rabbits developed HTSs gradually by full-skin defects (2 cm × 5 cm in diameter) that were created on the ventral side of ears. The rabbits were then randomly classified into four time groups (*n*=6 in each group) (see Table 1). The rabbits’ left ear of every group received UVA1 radiation at the same time—1, 2, and 3 months after the operations, respectively. The rabbits’ right ear was the control group. The animals were also divided into two dose subgroups (*n*=3 in each group) that received irradiation UVA1 for 30 sessions after the operations (two groups that received 60, 110 J/cm^2^/session, respectively).

**Table 1:** The classification of rabbits

Group	Control group (*n*=6)	Experimental group (*n*=24)			
		U1 (*n*=6) U1H (*n*=3);U1M (*n*=3)	U2 (*n*=6) U2H (*n*=3); U2M (*n*=3)	U3 (*n*=6) U3H (*n*=3); U3M (*n*=3)	U4 (*n*=6) U4H (*n*=3); U4M (*n*=3)
Methods	No operation on ears	UVA1 irradiation on the left ear 1 month after operation	UVA1 irradiation on the left ear 2 months after operation	UVA1 irradiation on the left ear 3 months after operation	UVA1 irradiation on the left ear while operation
UVA1 irradiation	No irradiation	H:110 J/cm^2^/session for 30 sessions M:60 J/cm^2^/session for 30 sessions	Like U1	Like U1	Like U1

Abbreviations: H, high-dose group; M, medium-dose group.

### Equipment

The UVA1 irradiation equipment consisted of UVA1 light device (Shanghai Sigma High-Technician Company, China) which emitted wavelengths exclusively in the 340–400 nm range. Frequency treatments were five times per week for six consecutive weeks. The operating voltage, frequency, and power of this equipment was AC 220 V, 50 Hz, and 300 V, respectively. The irradiance intensity was 65 mW/cm^2^.

### Measurement of scar with sliding caliper

Both the scar and neighboring normal skin thickness were measured with a sliding caliper before and after UVA1 treatment, respectively (precision was 0.02 millimeter). The scar index was equal to the ratios between the difference scar and neighboring normal skin thickness and normal skin thickness.

### Histopathology, immunohistochemistry and ultrastructure

Three millimeter punch biopsies were taken from the scar tissue before and immediately after UVA1 therapy respectively, except in the U4 group. The tissue of U4 group was obtained only post-treatment. The skin pieces were cut into half. One was fixed in 10% formalin solution and embedded in paraffin for standard histologic examination and immunohistochemical investigation and was stored in glutaraldehyde for transmission electron microscopic analysis. Consecutive 5-μm serial sections were cut and stained with Hematoxylin and Eosin. Dermal thickness was determined in Hematoxylin and Eosin-stained sections viewed under Axioplan 2 imaging analyzer (Shanghai Jiao Tong University, School of Medicine, Biochemical Department) and five randomly selected fields from each section of the rabbit skin. To determine the collagen content and organization of the lesional skin, deparaffinized sections were processed for Masson’s Trichrome stain, which was viewed under Axioplan 2 imaging analyzer (images were analyzed by digital capture). The ratio of positive stain counted in each field was then calculated. Ten random fields were selected in each section of rabbit skin and 5-μm sections from lesional skin samples were deparaffinized, re-hydrated in descending alcohol dilutions, and immersed in citrate buffer. To block the endogenous peroxidase activity, the sections were treated with peroxidase for 15 min at room temperature and then washed in phosphate buffer saline (PBS). The sections were stained using a standard avidine-biotin peroxidase technique with anti-transforming growth factor-β1 (1:100) (TGF-β1) polyclonal antibody (Wuhan Boster Biological Technology, Ltd.), MMP-1 (1:200) monoclonal antibody (Chemicon International, Inc.) TIMP-1 (1:100), polyclonal antibody (Chemicon International, Inc.); α-SMA antibody (α-SMA) (1:200) (Abcam). Three pictures were captured randomly. The ratio of positive stain counted in each field was then calculated.

### Statistical analysis

Results were expressed as means ± SD. Statistical analysis was performed with SPSS 11.0 for Windows. Significance testing was assessed by *t* test. *P*<0.05 was considered to be significant.

## Results

### Observations on cicatrical morphous in general

The raw surface in the rabbits ears were epithelizationed completely 20 days after the operation and the skin was thickened, stiffened and absent hair growth, whereas non-operation rabbit ears did not develop hyperplasia. The scar proliferated most obviously 3–4 months after the operation, but did not exceed the operation line. Part of the scar flattened 7–8 months after the operation, but remains of the scar existed. After different doses of UVA1 irradiation on different starting times, scar tissues were obviously softened. In comparison with pre-irradiation, the degree of cicatrix hyperplasia of the left rabbit ear (in groups U1, U2, and U3, which received irradiation UVA1 after wound surface healing) were improved. The HTSs were obviously induced in the rabbit ears of group U4, which received irradiation UVA1 while they were locally operated on.

### Scar index

In groups U1, U2, and U3, compared with pre-irradiation, the scar index was descended except U3M. Nevertheless, the scar index was elevated in the simultaneous irradiation group regardless of high or medium dose (Tables 2 and 4).

**Table 2:** Results of the skin thickness (S.T.), expressive rate of collagen (E.R.C.) and scar index (S.I.) before treatment (b.t.) and after treatment (a.t.) with medium- and high-dose ultraviolet A1 phototherapy*

Group	S.T. (μm)			E.R.C. (%)			S.I.		
	b.t.	a.t.	*P*-value	b.t.	a.t.	*P-*value	b.t.	a.t.	*P-*value
**U1H**	426.06 ± 72.55	282.32 ± 58.60	0.028*	81.30 ± 2.84	24.91 ± 16.88	0.023*	0.45 ± 0.06	0.28 ± 0.10	0.035 *
**U1M**	562.49 ± 133.46	523.09 ± 118.09	0.081	71.04 ± 6.57	44.51 ± 6.43	0.045*	0.40 ± 0.16	0.32 ± 0.15	0.043 *
**U2H**	478.94 ± 134.60	336.50 ± 98.34	0.039*	82.81 ± 3.96	34.47 ± 8.90	0.019*	0.67 ± 0.86	0.56 ± 0.10	0.019 *
**U2M**	627.22 ± 207.50	573.07 ± 177.52	0.096	67.97 ± 25.40	49.20 ± 28.78	0.015*	0.67 ± 0.06	0.57 ± 0.05	0.029 *
**U3H**	671.85 ± 139.94	525.13 ± 98.42	0.033 *	85.48 ± 3.74	58.34 ± 2.15	0.006*	1.19 ± 0.20	1.06 ± 0.23	0.023 *
**U3M**	560.43 ± 197.54	511.73 ± 165.83	0.119	81.98 ± 1.94	67.27 ± 6.88	0.057	1.19 ± 0.19	1.16 ± 0.18	0.085

* Data are reported as mean ± SD.

### Histologic analysis

**Figure 1:**
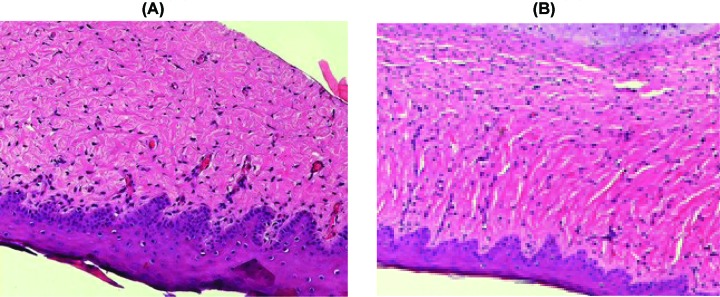
Histological changes before and after UVA1 treatment (**A**) The histopathological examination of the scar tissue prior to treatment showed atrophy of the epidermis, a low-grade inflammation and a dramatic loss of elastic tissue with thicker and more abundant collagen bundles in the dermis. (**B**) Biopsy from the treated side indicated marked decrease in the number of fibroblasts, and collagen fibers appeared looser, relaxed, and arranged in a random array, especially in the upper dermis (H&E, ×200).

Histologic evaluation of the biopsies revealed dense, coarse dermal fibrosclerosis devoid of skin appendages characterized by a low-grade inflammation with a small number of mast cells and a dramatic loss of elastic tissue with thicker and more abundant collagen bundles. Islands composed of aggregates of fibroblasts, small vessels, and thicker and stretched collagen bundles were seen throughout the dermis of HTSs before the UVA1 treatment (Figure 1A). After the UVA1 treatment, except in group U4, a decrease in the number of fibroblasts was seen, and collagen fibers appeared looser, relaxed, and arranged in a random array, especially in the upper dermis (Figure 1B). Compared with the pretreatment, no significant change in the number of tissue mast cells was observed after the UVA1 treatment. The collagen fibers appeared tighter, compacted, and arranged in an orderly array contrasted with the no-irradiation ear in group U4.

### Dermal thickness

Dermal thickness was determined by Hematoxylin and Eosin-stained sections. In comparison with pre-irradiation, the dermal thicknesses were decreased remarkably after high-dose UVA1 irradiation; nevertheless, there was no significant difference at medium dosage (Table 2). Contrasted with the control group (the right ears that did not receive UVA1 irradiation), the difference of dermal thickness in the left ears were statistically significant after high-dose UVA1 irradiation (Table 3). With group U4, (UVA1 irradiation on the left ear while operation), neither high- nor medium-dose could restrain the formation of a HTS, instead could increase the dermal thickness notably (Table 4). The effect of UVA1 on dermal thickness was dose-dependent in groups U1, U2, and U3 (Table 5).

**Table 3:** Results of the difference of skin thickness (S.T.) and expressive rate of collagen (E.R.C.) between pre-irradiation and post-irradiation with medium- and high-dose ultraviolet A1 phototherapy in treatment and control groups*

Group	S.T. (μm)			E.R.C. (%)		
	Treatment group	Control group	*P*-value	Treatment group	Control group	*P*-value
U1H	−143.52 ± 42.91	89.78 ± 25.29	0.027*	−56.39 ± 15.04	6.03 ± 4.03	0.028*
U1M	−54.15 ± 31.38	94.93 ± 93.00	0.138	−26.53 ± 10.11	5.79 ± 4.93	0.029*
U2H	−142.44 ± 49.96	304.94 ± 93.49	0.033*	−48.35 ± 10.44	18.13 ± 14.58	0.043*
U2M	−39.40 ± 20.63	133.60 ± 59.17	0.056	−18.77 ± 4.56	42.21 ± 16.83	0.021*
U3H	−146.72 ± 47.51	232.34 ± 86.21	0.010 *	−23.81 ± 6.88	30.03 ± 16.94	0.012*
U3M	−48.70 ± 32.06	80.40 ± 84.57	0.135	−14.70 ± 6.34	22.85 ± 14.18	0.030*

* Data are reported as mean ± SD.

**Table 4:** Results of the skin thickness (S.T.), expressive rate of collagen (E.R.C.) and scar index (S.I.) UV-treated ears and no UV-treated ears in U4 group with medium- and high-dose ultraviolet A1 phototherapy*

Group	S.T. (μm)			E.R.C. (%)			S.I.		
	UV	no-UV	*P*-value	UV	no-UV	*P-*value	UV	no-UV	*P*-value
U4H	811.68 ± 79.03	660.94 ± 107.96	0.019*	67.80 ± 9.06	38.42 ± 13.99	0.010*	0.88 ± 0.10	0.64 ± 0.05	0.023*
U4M	659.08 ± 178.98	394.37 ± 90.56	0.038*	61.35 ± 12.91	54.41 ± 11.80	0.037*	0.78 ± 0.07	0.63 ± 0.02	0.045*

* Data are reported as mean ± SD.

**Table 5:** Results of the difference of skin thickness (S.T.) and expressive rate of collagen (E.R.C.) between pre- and post-irradiation with medium- and high-dose ultraviolet A1 phototherapy in treatment group*

Group	The difference in S.T. (μm)			The difference in E.R.C. (%)		
	High-dose group	Medium-dose group	*P*-value	High-dose group	Medium-dose group	*P*-value
**U1**	−143.52 ± 42.91	−54.15 ± 31.38	0.044*	−56.39 ± 15.04	−26.53 ± 10.11	0.046*
**U2**	−142.44 ± 49.96	−39.40 ± 20.63	0.030*	−48.35 ± 10.44	−18.77 ± 4.56	0.011*
**U3**	−146.72 ± 47.51	−48.70 ± 32.06	0.041*	−27.14 ± 3.81	−14.70 ± 6.34	0.044*

* Data are reported as mean ± SD.

### Expressive rate of collagen

The expressive rate of collagen was determined by Masson’s Trichrome-stained sections. In comparison with pre-irradiation, both high- and medium-dose UVA1 could decrease the expressive rate of collagen in groups U1 and U2 remarkably (*P*<0.05). However, only high-dose UVA1 could degrade the expressive rate of collagen in group U3. Contrasted with the non-irradiated right ear, both high- and medium-dose UVA1 could decrease the expressive rate of collagen in groups U1, U2, and U3 remarkably. With group U4, both high or medium dose could increase the expressive rate of collagen significantly. The effect of UVA1 on the expression of collagen was dose-dependent in groups U1, U2, and U3 (see Tables 2–5).

**Figure 2:**
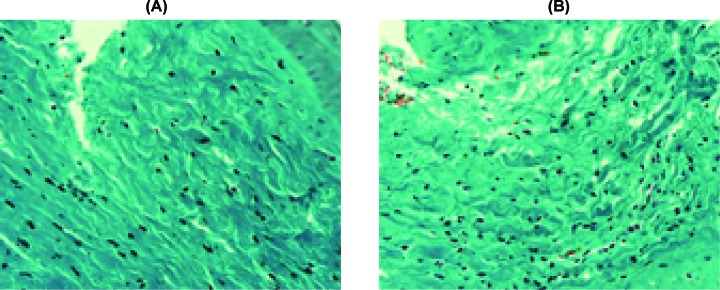
Changes of Masson’s Trichrome stain before and after UVA1 irradiation (**A**) The dermis was stained dark green to represent strong positive of collagen staining replacing the normal dermal architecture. (**B**) After UVA1 irradiation, there was less collagen deposition. The fibers appeared to be more loosely arranged, the collagen bundles appeared thinner, individually arranged and loose with widening of the spaces between them (Masson’s Trichrome stain, ×400).

**Figure 3:**
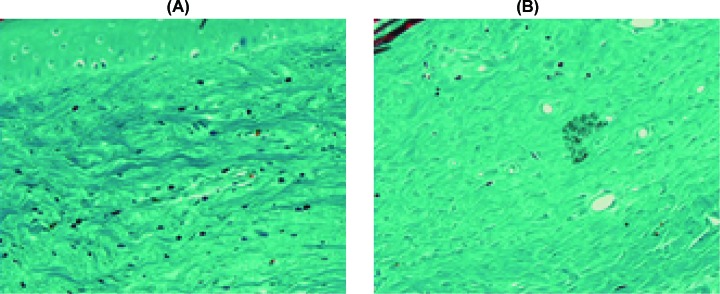
Changs of Masson’s Trichrome stain before and after UVA1 irratiation in group U4 (**A**) In group U4, strong staining in the dermis was noted in UVA1-treated rabbit ears. However, (**B**) revealed faint positive stain in not-received UVA1 irradiation ears, and the collagen bundles appeared thinner and looser (Masson’s Trichrome stain, ×400).

Masson’s trichrome stains collagen fibers green. As shown in Figure 2A, the dermis was stained dark green to represent a strong positive of collagen staining that replaced the normal dermal architecture. In contrast, there was less collagen deposition after UVA1 irradiation. The fibers appeared to be more loosely arranged. Following phototherapy, the collagen bundles appeared thinner, individually arranged and loose, with a widening of the spaces between them (Figure 2B). However, the staining outcome of U4 group was the opposite, namely, the collagen bundles appeared thicker and tighter compared with the control group (Figure 3).

### Immunohistochemical investigation

**Figure 4:**
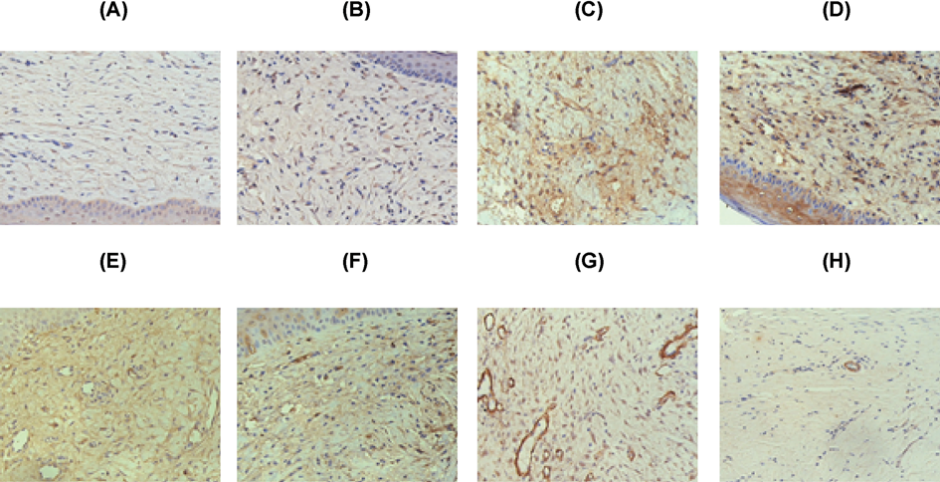
Effects of UVA1 treatment on immunohistochemistry To study possible mechanism of UVA1 phototherapy, skin biopsies were stained with anti-MMP-1 (**A,B**), anti-TIMP-1 (**C,D**), anti-TGF-β1 (**E,F**) and anti-α-SMA (**G,H**). The left panel represents biopsies before and the right panel shows biopsies after ultraviolet A1 treatment. In the present study, TIMP-1, TGF-β1 and a-SMA are decreased after therapy, whereas MMP-1 is increased. Specimens obtained before and after treatment were stained at the same time for the different antigens.

Quantitative staining of the specimens with a variety of antibodies against parameters of collagen metabolism revealed a decrease in the mean-staining intensity for tissue growth factor-β (TGF-β1) (both medium- and high-dose) and α-SMA (only U1H, U1M, and U2H groups), a decrease in TIMP-1 (only high-dose UVA1), and an apparent increase in the intensity for MMP-1 (both medium- and high-dose) compared with pre-treatment in groups U1–U3 (Table 6). An example of the staining patterns is shown in Figure 4.

**Table 6:** Results of quantitative immunohistochemical staining of skin specimens with antibodies to MMP-1, TIMP-1, TGF-β1, and α-SMA before treatment (b.t.) and after treatment (a.t.) with medium-dose ultraviolet A1 phototherapy*

Group	MMP-1			TIMP-1			TGF-β1			α-SMA		
	b.t.	a.t.	*P*-value	b.t.	a.t.	P value	b.t.	a.t.	*P*-value	b.t.	a.t.	*P*-value
**U1H**	5.30 ± 1.76	10.43 ± 1.61	0.006*	22.67 ± 4.00	12.74 ± 4.58	0.018*	19.90 ± 6.57	12.51 ± 4.13	0.042*	4.68 ± 1.18	1.33 ± 0.34	0.022*
**U1M**	4.06 ± 1.41	11.16 ± 1.57	0.004	24.77 ± 3.11	16.04 ± 2.07	0.095	17.64 ± 5.96	12.02 ± 5.02	0.032*	5.41 ± 0.77	2.04 ± 0.20	0.026*
**U2H**	3.08 ± 0.72	8.63 ± 2.61	0.037*	25.47 ± 2.15	15.17 ± 3.26	0.046*	31.24 ± 3.65	18.74 ± 6.42	0.017*	7.73 ± 0.82	3.60 ± 1.75	0.038*
**U2M**	3.46 ± 1.01	7.33 ± 1.58	0.048*	28.26 ± 1.37	20.06 ± 3.45	0.065	29.88 ± 4.65	19.69 ± 4.52	0.047*	8.19 ± 0.47	5.83 ± 0.87	0.069
**U3H**	2.63 ± 1.87	5.74 ± 1.43	0.034 *	29.19 ± 2.57	20.72 ± 3.31	0.007*	34.99 ± 4.91	20.51 ± 1.78	0.015*	9.58 ± 0.81	7.71 ± 0.83	0.054
**U3M**	2.16 ± 0.57	3.11 ± 0.27	0.037*	29.97 ± 7.27	23.77 ± 5.18	0.066	37.76 ± 4.14	29.45 ± 6.55	0.040*	10.31 ± 1.53	8.20 ± 0.23	0.108

* Data are reported as mean ± SD.

In group U4, it exhibited an increase in the staining for α-SMA and TIMP-1 in both high and medium doses. The expression of MMP-1 was depressed in the high-dose group, but was indistinctive in the medium-dose group. On the contrary, the expression of TGF-β1 was incremental in the high-dose group, but non-significant in the medium-dose group (Table 6).

### Ultrastructural analysis

**Figure 5:**
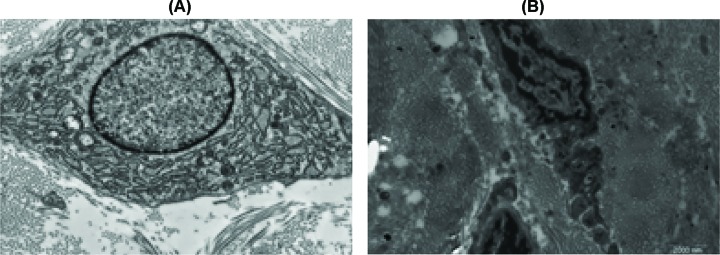
TEM results before and after UVA1 treatment (**A**) TEM prior to treatment showing enlarged endoplasmic reticulum (RER) and abundant cytoplasm in fibroblast (×23500 original magnification) (arrows indicate fibroblast). (**B**) TEM 6 weeks after treatment showing fibroblastic cytoplasm were lessened and most of them was in quiescent stage (×23500 original magnification).

**Figure 6:**
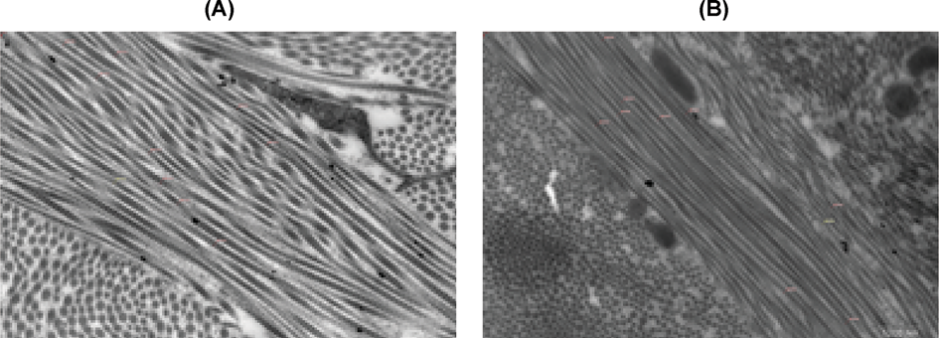
Changes of collagen fibers TEM post-treatment TEM post-treatment showing the collagen fibers diameter shrinking (**A**, pre-treatment; **B**, post-treatment) (×24500 original magnification).

Before treatment, transmission electron microscopic biopsies (TEM) confirmed the presence of enlarged rough endoplasmic reticulum (RER) of varying sizes and abundant cytoplasm in the fibroblast (Figure 5A). This was associated with an increased number of fibroblasts. Such findings are consistent with increased fibroblast activity during the development of HTS. After UVA1 treatment TEM showed the collagen fibers diameters shrinking, fibroblastic cytoplasm lessened, and most of them were in a quiescent stage (Figures 5B and 6). Such results coincide with the histological and immunohistochemical outcomes. Biopsies of random untreated normal skin in all the study subjects did not show such changes.

## Discussion

Studies published using UVA1 have demonstrated favorable improvements in scar bulk, pliability with minimal side effects, and treatment discomfort. However, few studies have reported that the effectiveness of UVA1 treatment is limited to alleviation of symptoms such as intense pruritus and this has not been mentioned with regard to the possible mechanisms. The mechanisms of UVA1 irradiation to improve HTS remains unknown. However, Petersen et al.’s study showed that UVA irradiation increased collagenase production by cultured fibroblasts [21] and it has been shown that increased collagenase expression in irradiated plaques of morphoea accompanies improvement with UVA1 phototherapy [10]. Perhaps the efficacy of UVA1 in morphoea, and in other conditions such as keloids and HTS, is partly due to the key role collagen proliferation plays in the early phase of HTS formation. Furthermore, conceivable theories also include fibroblast activity, collagen remodeling, and decreased cellular activity resulting from phototheraphy-induced localized tissue anoxia probably. Improvement in HTS might be related to not only one of the abovementioned mechanisms but also a combination of them. Our recent study has confirmed that clinical improvement of HTSs after UVA1 treatment is mirrored by histologic findings that showed a decrease in the number of fibroblasts and collagen fibers that appeared looser, relaxed, and arranged in a random array.

A model of HTS was induced by means of a full-skin defect on the ventral side of the ears. Histopathological examination was characterized by thicker and more abundant collagen bundles, deposition of homogeneous materials in the thickened dermis, and decreased hair follicles, and inflammatory infiltrates with cellular infiltrates that mimicked the histological features of human HTSs. It is hard to obtain the skin samples from scarred patients; therefore, in the present study, the rabbit ear model was selected for a classic model of HTS to investigate the conceivable effect of UVA1 in treating scars.

So far, various UVA1 sources are available including fluorescent lamp and high output metal halide. The fluorescent lamp cubicles allow only low (usually approximately 10–30 J/cm^2^) to moderate (40–70 J/cm^2^) individual treatment doses to be administered, whereas high-output metal halide sources (which require elaborate cooling systems in the case of whole-body treatment units) allow high doses (up to 130 J/cm^2^) to be administered during a single treatment session. In our study, the irradiance intensity of UVA1 equipment is 65 mW/cm^2^. The previous study involving localized scleroderma showed that [10] the fluence of UVA1 was held at medium dose (60 J/cm^2^) and high dose (110 J/cm^2^). Each single treatment takes approximately 15 and 30 min, respectively.

We further investigated the clinical efficacy of two doses of UVA1 (60, 110 J/cm^2^) and variable beginning irradiation times in the treatment of HTS of rabbit ears. Clinical improvement was evident as early as the third week of UVA1 treatment. After 6 weeks, remarkable softening and flattening were observed by three physicians. Although a relatively high dose (110 J/cm^2^) was adminstrated, no complication such as permanent pigmentary change, ulceration, or infection was seen. Histologically, after 6 weeks of post UVA1 treatment, the number of fibroblasts decreased and collagen fibers appeared looser, relaxed, and arranged in a random array, especially in the upper dermis compared with the pretreatment that was characterized by a dramatic loss of elastic tissue with thicker and more abundant collagen bundles throughout the dermis.

Our study demonstrated that scar improvement was significantly seen after receiving high-dose UVA1 beginning from 1 month of UVA1 irradiation. Additionally, the scar index and expressive rate of collagen were significantly reduced after the medium- and high-dose UVA1 treatment in U1 and U2 groups; only high-dose UVA1 irradiation reduced the scar index and expressive rate of collagen in U3 group. The effect of UVA1 on dermal thickness and expressive rate of collagen were in a dose-dependent manner. The better clinical results in rabbits with HTSs undergoing UVA1 therapy could be obtained when receiving high-dose UVA1 irradiation, which are consistent with the results in localized scleroderma patients with UVA1 treatment [10]. The most arresting finding is that neither high nor medium doses could restrain the formation of HTSs, even though they could increase the dermal thickness, expressive rate of collagen, and scar index notably. So the UVA1 irradiation could not restrain the formation of HTS rabbit ears model induced by a full skin defect if UVA1 interfered in the initial stage of wound healing.

Excessive deposition of connective tissue is the result of imbalance between synthesis and degradation. The metalloproteinases responsible for collagen cleavage have been characterized recently: MMP-1/collagenase 1 has been found to be involved in skin collagen degradation [22,23]. Disruption of the normal control of MMP-1 led to pathological consequences resulting from excessive accumulation or overdegradation of ECM [24]. Previous investigations demonstrated the UVA1-induced increase in specific messenger RNA of various MMPs (MMP-1, MMP-2, and MMP-3) in cultured human fibroblasts and the expression of MMP-1 mRNA was dose-dependent [25,26], which was then certified by other laboratories [10,27]. Simultaneously, an elevation of interstitial collagenase messenger RNA and protein expression could be determined immunohistochemically and by the use of nucleic-acid *in situ* hybridization in dermal fibroblasts [27]. Studies in patients with morphoea undergoing UVA1 therapy and *in vitro* experiments with fibroblast cultures have shown that MMP-1 as well as its specific inhibitor, TIMP-1, are activated following UVA irradiation [10,17,25–27].

Established models of photo damage in human skin propose an imbalance in this regulating circle that is responsible for remodeling UV-exposed skin and loss of collagen fibers [23].

In our investigation, we found markedly reduced TGF- 1 and TIMP-1 staining and enhanced MMP-1 staining when comparing the pre- and post-treatment biopsies in the immunohistochemical analysis, suggesting that a change in *de novo* synthesis after UVA1 therapy or, alternatively, an induction of collagen-degrading enzymes. The most intriguing finding is that the depression of TIMP-1 was dose-dependent, but the alteration of TGF-β1 and MMP-1 were dose-independent. As our results for TIMP-1 showed, only slight changes and MMP-1 production were visibly enhanced in medium-dose treatment, an exclusive impact of UVA1 on collagen degradation as the reason for diminished mature collagen in our subjects seems conceivable. The mild decrease in the dermal thickness but apparent decrease in the expressive rate of collagen after medium-dose UVA1 therapy could be accounted for only a slight change of TIMP-1 but a significant change of MMP-1 and TGF-β after medium-dose UVA1. However, we proposed that medium-dose UVA1 caused an initial strong stimulation of MMP-1 and lead to a breakdown of the collagen fibers that were present at a lower level, or that were balanced by the parallel induction of TIMP-1 at high-dose UVA1. This assumption would be in accordance with the findings of Gruss et al. [27] and Stege et al. [10] who described a rapid MMP-1-inducing capacity of UVA1 in the fibroblasts of morphoea patients as a relevant therapeutic effect.

It has been known that transforming growth factor-β (TGF-β) is a ubiquitous, multifunctional cytokine that plays an important role in regulating pro-collagen synthesis [28–33]. A wealth of evidence indicate that TGF-β1 plays a central role in controlling production of ECM proteins, and is critical for connective tissue regeneration during wound healing [34,35]. Interference with TGF-β1 expression in skin fibroblasts results in substantial reduction in type I pro-collagen gene expression, suggesting that autocrine production of TGF-β1 is primarily responsible for type I pro-collagen synthesis [36–40]. Additionally, overexpression of TGF-β1 in transgenic mice results in accumulation of type I collagen in skin connective tissue and other organs [41]. Taken together, these data indicate that TGF-β1 is a critical regulator of type I pro-collagen synthesis. TGF-β1 is also the major regulator of ECM synthesis in human skin, including stimulation of fibroblast proliferation in the dermis and down-regulation of expression of proteolytic enzymes such as collagenase and stromelysin [42]. In dermal fibroblasts, UV-induced impairment of the TGF-β/Smad pathway causes reduced production of type I pro-collagen, thereby leading to a loss of collagen [43]. TGF-β1 is an important fibrogenic cytokine that enhances collagen production, inhibits metalloproteinases and stimulates their inhibitors [23,44,45]. TGF-β1 was found to be excessively released by activated fibroblasts in morphoea and systemic sclerosis. Our data showed that UVA1 radiation not only regulated down expression of TGF-β markedly in rabbit *in vivo*, but also decreased dose-independently. Mofty et al. [46] and Yamane et al. [47] recently observed a significant down-regulation of TGF-β mRNA in patients with morphoea following a course of UVA therapy (total cumulative dose of 400 J/cm^2^). These observations give support to our results indicating that longer wavelengths in the UVA region are capable of down-regulating TGF-β/Smad signaling, particularly in the dermal compartment. Unlike shorter wavelengths in the UVB region, UVA1 radiation can penetrate into the deep dermis and is therefore capable of influencing TGF-β/Smad protein expression in dermal fibroblasts. Our data support recent studies indicating that UVA1 phototherapy is superior to UVB-based therapy in fibrotic skin conditions [48]. However, the most deserved cautious finding in our study is the decrease in MMP-1 and the increased expression of TIMP-1 and TGF-β1 in U4 group rabbit ears in the tissue specimens, which is opposite of the results of other groups. These results could explain the stronger increase in dermal thickness, scar index, and the expressive rate of collagen when treated with an operation and simultaneously received UVA1 radiation. Further investigation is required to confirm these results.

In recent years, it has gradually become clear that myofibroblasts are closely related to scar contracture [49,50]. Myofibroblasts are atypical fibroblasts, which have the characteristics of both fibroblasts and smooth muscle cells in ultrastructure. Myofibroblasts express high α-SMA levels and are responsible for fibrosis and tissue contraction via increased matrix synthesis. By secreting ECM proteins and promoting tissue contraction through the expression of a-SMA, myofibroblasts have been implicated in the pathogenesis of HTS. In our study, the myofibroblasts were identified by immunohistochemical detection of cytoplasmic a-SMA, and a marked decrease in a-SMA was observed in U1H, U1M, and U2H groups contrasted with pre-irradiation (Figure 4G,H), which indicated that UVA1 irradiation could depress the production of myofibroblasts through restraining the expression of a-SMA. We presumed that this could also be one of the mechanisms of UVA1 phototherapy effectiveness on initial HTS.

In conclusion, UVA1 irradiation decreases the dermal thickness, the expressive rate of collagen histologically, and suppresses collagen synthesis and myofibroblasts production through down-regulation of TGF-β1, TIMP-1 and a-SMA and up-regulates the expression of MMP-1 respectively, immunohistochemically resulting in clinically observed softening of scars and the improvement of scar index with measurement. UVA1 phototherapy is an effective and economical treatment that should be considered for the therapy of human HTSs. However, the scar would be aggravated if there is interference by UVA1 irradiation in the process of wound healing.

## Consent to Publish

All the authors have consented to publish this research.

## Ethics, Consent and Permissions

The study was carried out, in agreement with the guidelines for animal research, according to the Guide for the Care and Use of Laboratory Animals published by the ethics and research committee of our hospital.

## Abbreviations

ECM: extracellular matrix
HTS: hypertrophic scar
MMP-1: matrix metalloproteinase-1
TEM: transmission electron microscopy
TGF-β: tissue growth factor-β
TIMP-1: tissue inhibitor of metalloproteinase-1
α-SMA: α-smooth muscle actin
